# Midterm results of digastric trochanteric flip osteotomy for high acetabular posterior wall fracture

**DOI:** 10.1007/s00264-022-05446-6

**Published:** 2022-05-25

**Authors:** Yuneng Li, Yufeng Ge, Haonan Liu, Shiwen Zhu, Xinbao Wu

**Affiliations:** 1 Department of Orthopaedics and Traumatology, Peking University Fourth School of Clinical Medicine, Beijing Jishuitan Hospital, Beijing, China; 2 Department of Orthopaedics, Beijing Children’s Hospital, Capital Medical University, National Center for Children’s Health, Beijing, China

**Keywords:** Acetabular fracture, Posterior wall, Digastric trochanteric flip osteotomy, Kocher-Langenbeck approach

## Abstract

**Purpose:**

Kocher-Langenbeck (K-L) approach is widely used in surgery of posterior wall fracture of acetabulum. However, challenges are frequently encountered in fractures involving the superior dome due to its short of view. We aimed to evaluate the efficacy of digastric trochanteric flip osteotomy (DTFO) in the K-L approach for high posterior wall acetabular fracture (HPWF).

**Methods:**

From January 2014 to December 2016, 39 patients who suffered high posterior wall fracture (HPWF) were included in this retrospective study. All the patients were divided into two groups according to surgery type (17 standard K-L approach (control group), 22 with DTFO (DTFO group)). The Matta criterion was used to evaluate the accuracy of reduction according to post-operative CT image, while modified Harris hip score and 12-item short-form health survey (SF-12) were applied to measure the clinical outcomes.

**Results:**

The median follow-up period was 55 (45 to 62.5) months. Blood loss and operation time were similar between the two groups. DTFO group achieved much more anatomical reduction than the control group (54.6% vs. 35.3%; OR, 2.2; 95% CI, 0.6 ~ 8.08). Significantly better functional outcomes were found in the DTFO group (10% higher points than the control group, *p* < 0.05). All the patients receiving good-to-anatomical reduction ended with good or excellent outcomes. The total incidence of complications in the DTFO group was much lower than in the control group (40.9% vs. 70.6%, *p* = 0.07).

**Conclusions:**

Compared with the traditional K-L approach, the intraoperative DTFO technique enabled better quality of reduction in patients with HPWF, thus ensuring superior clinical outcomes.

## Introduction

Posterior wall acetabular fracture is the most common acetabular fracture type, accounting for about 24% [1]. High-energy trauma is the primary cause. In recent years, with the rapid industrial development, traffic injuries have been increasing significantly and so does the incidence of acetabular posterior wall fracture-dislocation [2]. In such a situation of acetabulum injury, the acetabular roof, which is responsible for weight-bearing and of paramount importance for hip stability, can usually be jeopardized. As high as 76% of the posterior acetabular wall fractures reportedly involved the superior acetabular dome, defined as a high posterior wall fracture (HPWF) [3]. Failed reconstruction or any disruption of the intactness of such a load-bearing roof could directly alter the force distribution and then accelerate the degeneration of the articular cartilage, which is tightly associated with traumatic arthritis and poor functional outcomes [4]. Therefore, to optimize the continuity and smoothness of the roof, open reduction and internal fixation (ORIF) has been recognized as the treatment of choice since the last century, and Kocher-Langenbeck (K-L) approach has become the most common way to tackle HPWF [5].

However, it is recognized that the K-L approach has limitations regarding surgical field exposure [6]. When the fracture lines involve the anterolateral aspect of the acetabulum or a comminuted pattern of the roof, the standard K-L approach is short of bringing a full visualization of the acetabulum to the surgeons. In recent years, the digastric trochanteric flip osteotomy (DTFO) technique used in the K-L approach was introduced with good merits in expanding the field, facilitating ORIF, and minimizing the impact on the abductor strength after surgery [7]. Naranje et al. [8] also pointed out that compared with traditional trochanteric osteotomy, DTFO led to less compromise on the blood supply of the femoral head. Gupta et al. [9] verified its advantage in tackling problems involving the acetabular roof, but also raised their concern that a high risk of potential complications and extended operation time may occur. As far as we know, few studies have compared the standard K-L approach and combined DTFO approach, leading to limited evidence to discuss the pros and cons of such techniques.

In this presented study, we reviewed 39 HPWF patients with posterior dislocation treated with either combined DTFO or a standard K-L approach to explore the impact of the DTFO technique. Our prespecified hypothesis is that the combined DTFO approach can achieve a better quality of reduction and better clinical outcomes than the standard K-L approach.

## Materials and methods

### Design

**Fig.1:**
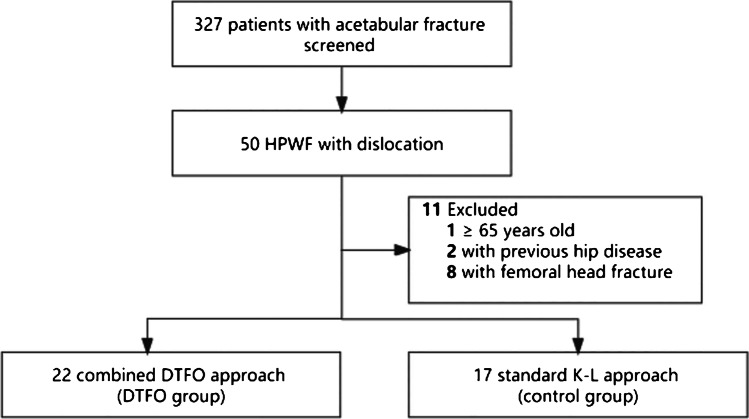
Flowchart of study. HPWF, high posterior wall fracture; K-L, Kocher-Langenbeck; DTFO, digastrics trochanteric flip osteotomy

**Fig.2:**
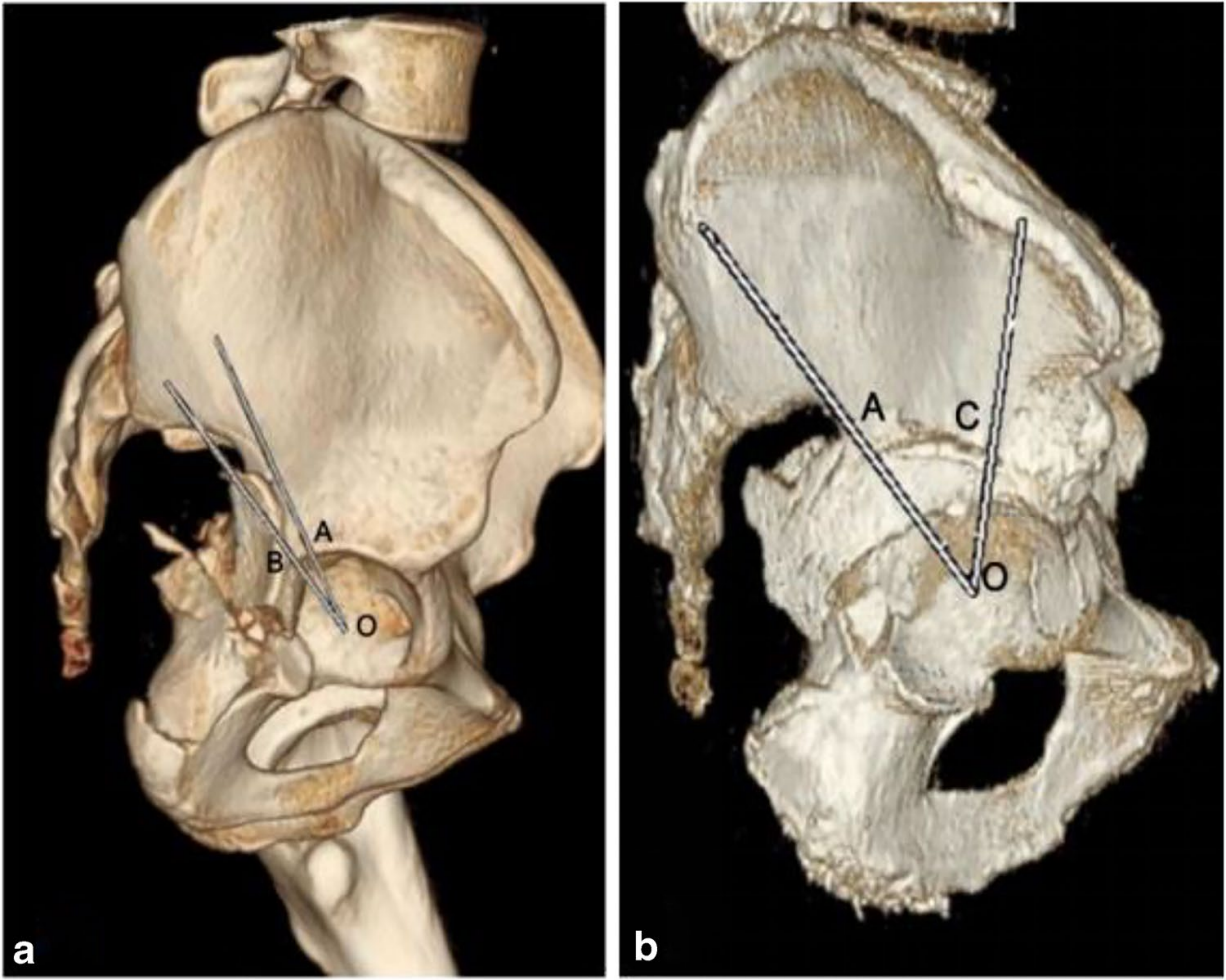
Method for determining the high posterior wall fracture (HPWF). O, centre of the acetabulum; B/C, upper end of the wall fracture line; A, apex of the greater sciatic notch

Between January 2014 and December 2016, 327 patients diagnosed with acetabular fracture and admitted for surgical treatment in our hospital were retrospectively screened. After reviewing the clinical records and radiographs, we only included those with high posterior wall fracture-dislocation treated with the standard K-L approach or the combined DTFO approach (Fig. 1). According to the method described by Harnroongroj [3], with the help of 3D computer tomography (CT) reconstruction, if the line drawn from the acetabular centre to the upper end of the acetabular fracture appears above the centroacetabulo-greater sciatic notch line, it will be regarded as involving the superior acetabular dome, namely, the HPWF (Fig. 2). The exclusion criteria included open fractures, the time interval from injury to surgery beyond two weeks, severe concomitant lower extremity fracture at the ipsilateral side (like pelvic fracture, femoral head, or neck fracture), previous hip diseases (like osteoarthritis, necrosis of femoral head), and previous hip surgery. Patients who could not walk independently or had difficulty taking care of themselves before the injury were also ruled out.

Patients were divided into two groups according to the surgical approach (combined DTFO approach as the DTFO group, standard K-L approach as the control group). Selection of the DTFO approach primarily depended on the potential difficulties during intra-operative visualization, reduction, and fixation. Surgeons’ familiarity with such an approach was also taken into account. Data regarding demographic information, injury mechanism, associated injury, deep vein thrombosis (DVT), presence of the comminution or impaction of the fractured wall, and time elapsed from injury to the operation were collected. Perioperative information was also documented, such as the American Society of Anesthesiologists (ASA) score, operation time, and intra-operative blood loss.

This retrospective study was approved by our institutional ethics committee and was conducted following the latest version of the Helsinki Declaration. All patients were informed about the study and signed an informed consent form.

### Outcome measures

Routine X-rays (anteroposterior and two Judet radiographs) and CT scan were arranged on the second day after surgery and used to evaluate the accuracy of reduction. The quality of reduction was assessed based on measurements of the greatest residual displacements on both X-rays and CT images according to the criteria of Matta [10] (0–1 mm as anatomical, 2–3 mm as good, > 3 mm as poor). Two observers who were blinded to the clinical outcome and uninvolved in the initial surgical care of the patients assessed the post-operative radiographs and CT scans in consensus. The functional outcome was evaluated with a modified Harris Hip Score (mHHS) [11], which was reported to be more representative in the acetabular fracture patient series. This scoring system consists of four fields (pain, function, relative strength, and relative range of movement), ranging from 0 to 120 (the higher score, the better function). Concerning the health-related quality of life (HRQoL), the 12-item short-form health survey (SF-12) [12] was used, which is comprised of two dimensions (physical component summary (PCS-12) and mental component summary (MCS-12)) that measure eight health domains. X-rays and physical examination during follow-up were used to check and document the complications, including fixation failure, decreased abductor strength, traumatic arthritis, and heterotopic ossification (HO). CT and MRI were also employed when appropriate. Brooker [13] classification was used to grade the HO and Medical Research Council (MRC) grading system for the abductor power [7].

### Surgical technique in K-L approach combined with DTFO

**Fig.3:**
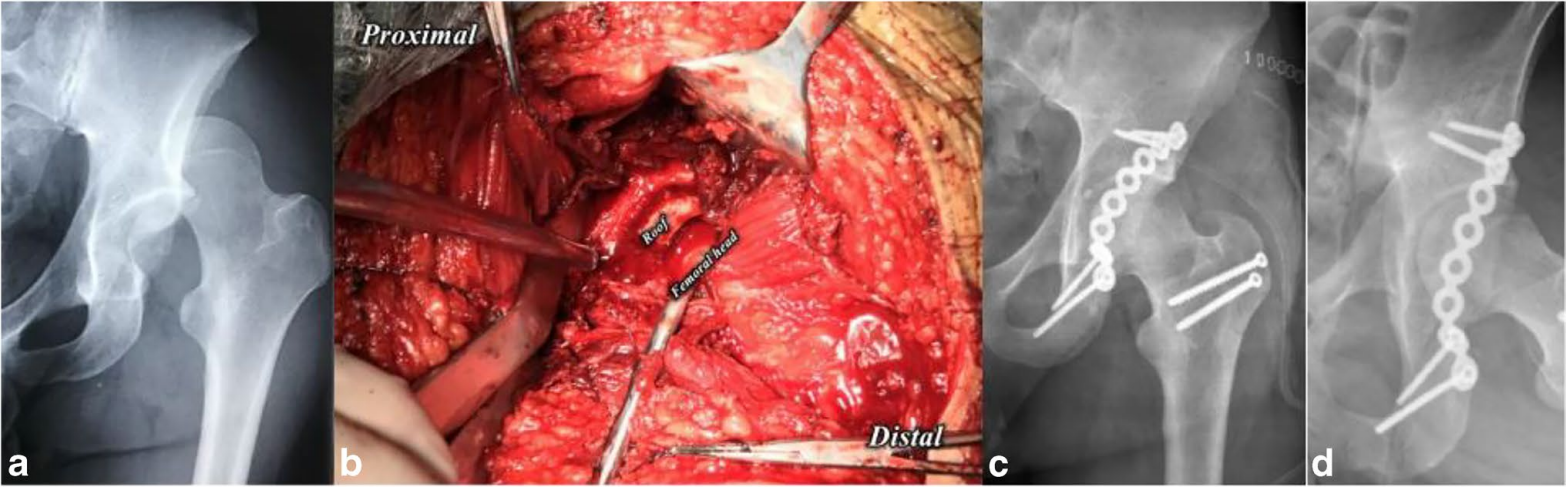
Middle-aged male with posterior wall fracture-dislocation after a traffic injury, treated via combined DTFO approach. **a** Left posterior wall fracture-dislocation; **b** Intra-operative image, DTFO help fully visualize the involved roof; **c**–**d** post-operative radiographs showing anatomic reduction

Siebenrock [7] described such a technique in his published study. Among our present cases, all patients were placed in a prone position, and the typical incision for a standard K-L approach was used. After carefully splitting the fascia latae and the gluteus maximus muscle, the trochanteric bursa is longitudinally incised, and the posterior borders of the gluteus medius muscle and the vastus lateralis muscle were then identified. The vastus lateralis muscle is detached from the femur at its posterior insertion over 5 to 10 cm distal to its origin at the vastus lateralis ridge. Next, an oscillating saw or an osteotome was used to conduct the digastric trochanteric osteotomy in a sagittal plane, with the gluteus medius muscle and the entire origin of the vastus lateralis muscle still attached to the medallion of the greater trochanter. The thickness was about 1.5 cm. During osteotomy, close attention should be paid to the deep branch of the medial circumflex artery crossing posterior to the obturator externus. All external rotator insertions remained on the proximal femur. After osteotomy, the gluteus minimus muscle and the piriformis tendon were dissected from the underlying bone and capsule, following which the osteotomized fragment can be rotated and retracted anteriorly. Siebenrock [7] described this procedure as resembling a patella in a standard approach to the knee joint, and flexing and externally rotating the femur may facilitate this. Next, capsulotomy was conducted to visualize the hip joint, and care should be taken not to damage the acetabular labrum (Fig. 3). Sometimes, anterior or posterior dislocation of the femoral head may be required to help remove the intra-articular fragment or directly assess the reduction of comminution walls. In such situations, the vascularity of the femoral head could be confirmed with prompt bleeding after drilling or pricking. At the end of the surgery, all the flip osteotomies were fixed using two or three cortical or cancellous screws (Fig. 3).

### Data analysis

Data are presented as means and standard deviations for parametric data or as medians and interquartile ranges when the data are not normally distributed. Categorical variables are described using frequencies and numerical distributions. The chi-squared test was used to assess the differences between two groups for categorical variables and Student’s *t*-test or the Mann–Whitney *U*-test for continuous variables, as appropriate (parametric vs. non-parametric data, respectively). Linear regression analysis was used to adjust the confounders associated with the outcomes (mHHS, PCS).

The analyses were performed with the statistical software packages R 4.1.1 (http://www.R-project.org, The R Foundation) and Free Statistics software versions 1.4. A two-tailed test was performed, and *p* < 0.05 was considered statistically significant.

## Results

From January 2014 to December 2016, 39 patients (13 females, 26 males; mean age, 40.9 years; age range, 19 to 64 years) were ultimately compared in our study. The flow is shown in Fig. 1. Of them, 22 patients (15 males) were treated by the combined DTFO approach and 17 (11 males) by the standard K-L approach. The baseline characteristics were similar and comparable between these two groups (Table 1). In both groups, nearly half of the patients sustained association injuries. All the chest and abdomen injuries were considered symptomatic treatment. Two patients in each group had shown dysfunction of the sciatic nerve before surgery and spontaneously recovered within six months after surgery.

**Table 1:** Baseline characteristics of patients in two groups

Characteristics	Control group *n* = 17	DTFO group *n* = 22	*p* value
Age(years), mean ± SD	39.3 ± 11.9	42.1 ± 13.6	0.505
Gender			1
Female	6 (35.3%)	7 (31.8%)	
Male	11 (64.7%)	15 (68.2%)	
Height(cm), mean ± SD	169.5 ± 8	169.5 ± 8.5	0.995
Weight(kg), mean ± SD	66.4 ± 16.7	69.8 ± 12.4	0.458
BMI(kg/m.^2^), mean ± SD	22.8 ± 4.1	24.2 ± 2.6	0.231
Pre-operative ASA			0.465
I	5 (29.4%)	4 (18.2%)	
II	12 (70.6%)	18 (81.8%)	
Comminution or impaction	7 (41.2%)	7 (31.8%)	0.789
Injury mechanism			0.95
Traffic injury	13 (76.5%)	17 (77.3%)	
Fall from heights	4 (23.5%)	5 (22.8%)	
Associated injury	7 (41.2%)	10 (45.5%)	1
Thoracic trauma	3 (17.7%)	2 (9.1%)	
Abdomen trauma	1 (5.9%)	3 (13.6%)	
Fracture of the vertebrae or extremities	3 (17.7%)	5 (22.7%)	
DVT	4 (23.5%)	6 (27.3%)	1
Time from injury to operation(days), median (IQR)	6.0 (5.0, 7.0)	6.5 (5.2, 9.0)	0.230

*BMI* body mass index, *ASA* American Society of Anesthesiologists score, *DVT* deep vein thrombosis

### Operation-related outcome comparison

As shown in Table 2, patients in the DTFO group received much more anatomical reduction than the control group (54.6% (12/22) vs. 35.3% (6/17); odds ratio (OR), 2.2; 95% CI, 0.6 ~ 8.08, *p* = 0.235), although no statistical significance was seen. With respect to operation time and intra-operative blood loss, the DTFO group saved about 15% time and caused extra blood loss, but still, the differences were not statistically significant.

**Table 2:** Operative data and outcomes compared between two groups

Outcomes	Control group *n* = 17	DTFO group *n* = 22	*p* value
Operation time (min), mean ± SD	159.2 ± 53.7	134.7 ± 33.2	0.23
Intra-operative blood loss (ml), mean ± SD	629.2 ± 291.5	713.2 ± 345.5	0.12
Hospital stays (d), mean ± SD	13.1 ± 3.7	15.1 ± 3.1	0.25
Complications	12 (70.6%)	9 (40.9%)	0.129
Internal fixation failure	2 (11.8%)	0 (0%)	
Decreased abductor strength	4 (23.5%)	3 (13.6%)	
Traumatic arthritis	2 (11.8%)	1 (4.6%)	
Heterotopic ossification	3 (17.7%)	5 (22.7%)	
Quality of reduction			0.563
Anatomical	6 (35.3%)	12 (54.6%)	
Good	9 (52.9%)	9 (40.9%)	
Poor	2 (11.8%)	1 (4.6%)	
mHHS, mean ± SD	98.8 ± 15.6	107.9 ± 10.6	0.037
PCS, mean ± SD	46.4 ± 7.9	51.8 ± 5.4	0.016
MCS, mean ± SD	58.0 ± 2.3	58.9 ± 2.3	0.253

*mHHS* modified Harris Hip Score, *PCS* physical component summary, *MCS* mental component summary

### Mid-term outcomes and complications

All the patients were followed up for at least 40 months (median 55 (45 ~ 62.5) months). Patients in the DTFO group yielded significantly higher mHHS than those in the control group (107.9 ± 10.6 vs. 98.8 ± 15.6, *p* = 0.037) (Table 2). Regarding HRQoL, both PCS and MCS favoured the DTFO group, although the significant between-group difference only lay in the physical behaviours (*p* = 0.016, Table 2). After adjustment, the trends were still significant (Table 3 in the Appendix).

**Fig.4:**
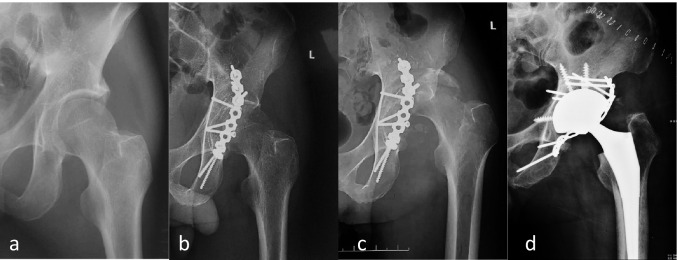
Case of a 45-year-old male with high posterior wall fracture-dislocation failing fixation after surgery with standard K-L approach. **a** Left high posterior wall fracture-dislocation (after closed reduction in other hospital); **b** open reduction and internal fixation using standard K-L approach; **c** re-dislocation within 2 months after surgery; **d** secondary total hip arthroplasty

As to complications, the total incidence in the DTFO group was relatively lower than the control group (40.9% (9/22) vs. 70.6% (12/17); OR, 0.29; 95%CI, 0.08 ~ 1.11, *p* = 0.07) (Table 2). Two patients in the control group failed the fixation and experienced re-dislocation of the affected hip within three months after surgery, and secondary total hip replacements were subsequently performed (Fig. 4 in the Appendix). The decreased abductor strength in both groups was given physiotherapy and gradually restored during the follow-up period. Only one patient in the DTFO sustained class IV HO resulting in a poor functional outcome, while the other patients with HO showed only mild radiograph changes (Brooker class I or II) without clinical significance. All the osteotomy sites in the DTFO group achieved uneventful union.

## Discussion

Posterior acetabular wall fracture-dislocation involving the superior dome remains a challenging field for any orthopaedic surgeon [1, 2]. Adequate intra-operative exposure and minimal damage to the surrounding soft tissue are prerequisites for anatomic reduction and decent prognosis [1, 5]. In our presented study, we compared the combined approach (K-L approach with DTFO) with the most widely used K-L approach to explore the role of the DTFO. Patients in the DTFO group were more likely to achieve anatomic reduction and yield fewer complications. During mid-term follow-up, the DTFO group scored about 10% higher points than the control group with respect to functional outcomes (mHHS and PCS) (*p* < 0.05, Table 2).

To the best of our knowledge, no comparative studies were conducted to test whether there exists a level of superiority of DTFO used in the K-L approach, although such a technique was introduced several decades ago and described as effective in certain types of acetabular fracture. Our study focused on a specific subgroup of patients with HPWF and dislocation and became the first comparative study to explore the impact of DTFO on mid-term outcomes.

Matta’s largest outcome study [14] of operatively treated acetabular fracture concluded several risk factors of unfavourable early prognosis, most of which were presented in our cohort. Age, dislocation, posterior wall involvement, marginal impaction, and the initial displacement were independent noncontrollable factors in our study. The most vital factors that were controllable by the surgeon, described by Matta [14], were the accuracy of reduction, which was also widely recognized as the determining one that decided the functional outcome [5, 14–16]. In our present study, a much higher anatomical reduction rate could be seen in the DTFO group (54.6%) compared with the control group (35.3%). We believed a reasonable explanation behind this was that the DTFO provided an extensive visualization of the involved acetabular wall and thus allowed relatively convenient access to the direct reduction of displaced fragments. In our clinical experience, sufficient exposure, particularly in intra-articular fracture, is the key to a successful ORIF [10]. The osteotomy technique we described was also proved effective in other studies. Siebenrock et al. [7, 17] reported 80% (8/10) and 83% (10/12) anatomical reduction rates in their two studies. Tannast [18] described a dramatically high anatomical reduction rate of 93% (50/54) in their study using DTFO to facilitate hip dislocation for acetabular fractures. The anatomical reduction rates in our study seemed lower than that reported in other literature [8, 14, 16–19]. Perhaps it was related to the method in which multiplanar reconstruction CT images were employed rather than X-rays, revealing more accurate details [20]. Overall, we achieved 90% good-to-anatomical reductions in both groups.

In our present study, the DTFO group yielded much better functional results and scored much higher physical points than the control group (Table 2). After establishing various multivariate regression models (Table 3 in the Appendix), we found that such a significant between-group difference primarily resulted from the gap in reduction accuracy mentioned above. In addition, the mHHS system used in our study was a modified version that helped improve the discrimination and detect easier the differences in treatment outcome of acetabular fractures. As far as we know, there are limited studies that discussed the clinical results of patients with posterior wall fracture-dislocation and employed the mHHS outcome. However, consistent with the results described in other literature using DTFO technique [18, 21, 22], about 90% of the DTFO group patients in our study achieved good-to-excellent functional outcomes with 85 points as the good threshold. Naranje et al. [8] reported a mean Merle d’ Aubigné score of 17.6 out of 18 points, while Masse [21] reported 15 points. Moed et al. [19, 23, 24] conducted a series of studies of patients with posterior wall fracture and found similar results that about 90% of patients achieved good-to-excellent results evaluated at a mean of five years after surgery. In our study, we are not surprised that all the patients receiving good-to-anatomical reduction ended up with good or excellent outcomes.

Two control group patients in our cohort failed the reduction and fixation. It is plausible that overlooking or inability to address the jeopardized superior dome would weaken the whole stability and thus lead to failure when an external load is applied like sitting. No femoral head necrosis or nonunion of the osteotomy site occurred in the DTFO group, and the abductor strength seemed less compromised than in the control group. As described by other researchers [7, 8], the assistance of DTFO enjoyed less vigorous retractions and minor iatrogenic damage to the abductors than the standard K-L approach initially introduced by Letournel and Judet [5]. Reduced muscle stripping could help maintain the abductor strength and minimize the HO. The patients in DTFO group were less likely to suffer decreased abductor strength, although all the strength compromise was restored after physiotherapy at the last follow-up. In our cases, prophylactic medication consisting of indomethacin 25 mg orally three times a day for three weeks was given to all patients to prevent HO. In terms of operation time and blood loss, we did not find a significant discrepancy between the two groups. The average time in both groups was less than three hours, somewhat lower than the reported results [7]. Perhaps the saved time during reduction and fixation in the DTFO group was offset by the additional procedure like osteotomy and fixation of the osteotomized fragment. Another fundamental reason may be that all the operations were performed by senior orthopaedic surgeons experienced in managing acetabular fractures.

The potential strengths of our study were its relatively large sample size due to the rarity of HPWF and comparative analysis rather than simply a descriptive report. Also, our trial possessed several limitations. First, the information we collected was limited due to its retrospective nature. Thus, records such as the cartilage damage of the femoral head and post-operative rehabilitation and the indications for DTFO could not be precisely documented. Further prospective research evaluating all the risk factors may identify the predictors of the prognosis in patients with HPWF and explore which kind of patients could benefit from DTFO approach. Second, early necrosis of the femoral head could not be easily spotted through X-rays or CT scan. Long-term follow-up or magnetic resonance imaging may be required to detect it.

In conclusion, compared with the traditional K-L approach, the intra-operative DTFO technique enabled better quality of reduction in patients with HPWF, thus ensuring superior clinical outcomes. In the future, prospective-designed comparative studies with larger samples may be required to explore the merits and indications of DTFO in acetabular fracture.

## Data Availability

The datasets used in this study are not publicly available because of patient confidentiality but are available from the corresponding author on reasonable request.

## Appendix

Fig. 4

Table 3

**Table 3:** Multivariable linear regression of the mHHS and PCS between two groups

	*β* value (95% CI)^*^	*p* value
mHHS		
Crude	9.09 (0.86 ~ 17.31)	0.037
Model 1	9.46 (1.26 ~ 17.66)	0.03
Model 2	9 (0.25 ~ 17.75)	0.052
Model 3	7.71 (− 1.19 ~ 16.61)	0.101
PCS		
Crude	5.41 (1.22 ~ 9.59)	0.016
Model 1	5.67 (1.54 ~ 9.8)	0.011
Model 2	5.54 (1.18 ~ 9.89)	0.018
Model 3	4.62 (0.39 ~ 8.85)	0.042

Model 1, adjusted for age + gender; model 2, adjusted for model 1 + associated injury + time from injury to operation + comminution or impaction; model 3, adjusted for model 2 + quality of reduction; *mHHS* modified Harris Hip Score, *PCS* physical component summary, *MCS* mental component summary

^*^Regard control group as the reference
